# Determinants of Physical Activity in Older Adults in South-Eastern Poland

**DOI:** 10.3390/ijerph192416922

**Published:** 2022-12-16

**Authors:** Agnieszka Wiśniowska-Szurlej, Agnieszka Ćwirlej-Sozańska, Anna Wilmowska-Pietruszyńska, Bernard Sozański

**Affiliations:** 1 Institute of Health Sciences, College of Medical Sciences of the University of Rzeszow, University of Rzeszow, 35-310 Rzeszow, Poland; 2 Faculty of Medicine, Lazarski University, 02-662 Warsaw, Poland; 3 Institute of Medicine, College of Medical Sciences of the University of Rzeszow, University of Rzeszow, 35-310 Rzeszow, Poland

**Keywords:** physical activity, aged, health status, ICF

## Abstract

The aim of our study is to assess factors determining the uptake of physical activity (PA) by older people living in south-eastern Poland. This is a cross-sectional study. The study included 858 older people aged 75 and over living in south-eastern Poland. PA was assessed by asking about the time spent on any at least moderate PA per week and about doing planned strengthening exercises to improve muscle strength and muscular endurance. Functional status, disability and quality of life in older people were also assessed. Logistic regression models were used to identify the factors related to PA. In the study group, only 25.64% performed a minimum of 150 min of moderate-intensity exercise, while strengthening exercises were performed by 22.49%. The most important factors influencing the uptake of PA were age, number of chronic diseases, place of residence, education, social activity, housing conditions, quality of life and health status. In summary, the study population represents a low level of PA uptake, with the majority not meeting the World Health Organization recommendations for PA uptake by older people. Our findings suggest individualized efforts to promote public health and increase PA among older people over 75 years of age.

## 1. Introduction

Low levels of physical activity (PA) are one of the major risk factors for mortality from non-communicable causes worldwide [1]. More than 31% of people globally are insufficiently physically active [2]. In Poland, older people are characterized by low levels of PA. According to the Polish Central Statistical Office (GUS), only one in four seniors undertakes recreational PA (25.1% of people) [3].

Regular PA in older people has a number of health benefits, such as reducing the risk of cardiovascular disease, functional limitations, cognitive impairment and improving wellbeing and mental state [4,5,6]. PA also plays an important role in the treatment and management of chronic diseases, e.g., hypertension, hyperlipidemia, type 2 diabetes and obesity [4]. High PA reduces overall mortality by 22–34% and chronic disease mortality by 27–35% [7].

The World Health Organization (WHO) recommends that older people engage in moderate-intensity aerobic PA of at least 150 min per week or vigorous aerobic PA of at least 75 min per week [8]. However, in many countries, the indicated recommendations are not being met [9], and the number of inactive people increases with age, reaching almost half of the population for those over 80 years of age [10].

Individual factors influencing the uptake of physical activity by older people are variable and include an individual′s psychological characteristics, attitudes, motivations and beliefs, as well as socio-demographic characteristics such as age, gender or level of education [11,12]. In contrast, social factors influencing PA include social support, social networks and neighborhood factors [13].

In 2018, the WHO established the Global Action Plan on Physical Activity 2018–2030, which sets out policy actions to increase PA levels among people around the world. The WHO′s main goal is to reduce physical inactivity by 15% by 2030, calling on national policies to build targeted strategies to promote PA among older people.

The specific sociodemographic characteristics of the population should be considered by local governments when shaping PA policies aimed at increasing activity among seniors. To effectively promote PA and plan supportive interventions, it is essential to analyze the factors influencing the uptake of physical activity by older people. Therefore, the aim of our study is to assess the determinants of physical activity in older people living in south-eastern Poland.

## 2. Materials and Methods

### 2.1. Study Design and Participants

This is a cross-sectional study. The study included 858 older people aged 75 and over living in south-eastern Poland. Sample size calculation was carried out based on the following assumptions: a 95% (0.95) confidence level and the size of the fraction of older people not undertaking physical activity of 0.8 [14], with a maximum estimation error of 3%. Inclusion criteria for the study group were: age 75 years or older, normal cognitive status (AMTS—Abbreviated Mental Test Score > 6 points) and informed consent to participate in the study. Exclusion criteria were: age below 75 years, cognitive impairment (AMTS ≤ 6 points) and a lack of consent to participate in the study.

The study was conducted in 2017–2018 by appropriately prepared and trained interviewers in the form of face-to-face interviews carried out using the Pen and Paper Interviews method at the respondents′ place of residence.

### 2.2. Data Collection

An abbreviated version of the Abbreviated Mental Test Score (AMTS) questionnaire was used to assess cognitive status [15].

Using an interview questionnaire, information concerning socioeconomic variables was collected, such as: age, gender, place of residence, marital status, education and income.

#### 2.2.1. Physical Activity Assessment

Physical activity was assessed by asking about the time spent on any at least moderate PA per week (resulting in at least light breathlessness, sweating, fatigue) and doing planned strengthening exercises to improve muscular strength and endurance according to the recommendations of the European Network for Action on Aging and Physical Activity [16].

#### 2.2.2. Health Status Assessment

Codes from the WHO International Classification of Functioning, Disability and Health (ICF) checklist were selected to investigate various factors influencing PA uptake among older people in Poland [17]. A literature review was conducted, and relevant questions were mapped for the selected categories according to the WHO recommendations [18]. Questions were developed for the selected ICF codes, the reproducibility and relevance of which were assessed at the pilot study stage.

The following physical health information was collected: height and weight (including BMI calculations, which were assigned to the ICF code B530 weight maintenance functions), incidence of pain (ICF B280 Perception of pain) and number of chronic diseases, as a quantitative variable related to health status as a general reference to the ICD-10 classification. Information was also collected on social activity and participation, including participation in social groups or organizations (ICF d910 Community life), and maintaining good relationships with relatives (ICF d760 Family relationships) and friends (ICF d720 Complex interpersonal interactions). Survey participants were also asked about their living environment, including the existence of barriers and obstacles (including architectural, communication, social and other barriers) (a comprehensive selection of environmental factors included in the Shorter Environmental List on the WHO ICF checklist) and their housing conditions (ICF e155 Design, construction and building products and technology of buildings for private use).

#### 2.2.3. Disability Assessment

Functioning and health was assessed using the 36-item WHODAS 2.0. The questionnaire measures general disability and disability in six domains: Do1 Cognition (6 items), Do2 Mobility (5 items), Do3 Self-care (4 items), Do4 Getting along (5 items), Do5 Life activities (8 items) and Do6 Participation (8 items). During the interview, the response refers to the last 30 days. Answers to the questions are rated on a 5-point scale identifying the level of difficulty or problem (1 = none, 2 = mild, 3 = moderate, 4 = severe, 5 = extreme or cannot do). The obtained results were converted on a scale from 0 to 100 [19].

#### 2.2.4. Quality of Life Assessment

Quality of life was assessed using the World Health Organization Quality of Life (WHOQOL) scale [20]. The WHOQOL is used to assess the following domains of quality of life: physical functioning, mental functioning, social functioning and environmental functioning. The answers to the questions were rated on a five-point scale that determines the level of difficulty or problem. The scale is oriented positively, which means that a higher number of points denotes a better quality of life (points 1–5) [21].

### 2.3. Statistical Analysis

For the analysis, a breakdown was adopted: people meeting the WHO recommendations for physical activity, i.e., undertaking moderate-intensity PA for a minimum of 150 min per week, and performing muscle-strengthening exercises. The cut-off point for assessing PA was established on the basis of the WHO recommendations [16].

Sociodemographic data and factors related to health status are presented as descriptive statistics. Assessments of disability and health status and quality of life in relation to PA levels are also presented. For quantitative variables, the mean and standard deviation are given and for categorical variables, the quantities and percentages are given. The chi-square test (for categorical variables) and the Mann–Whitney test (for quantitative variables) were used for a preliminary analysis of the association between individual demographic variables and health status factors and physical activity uptake. The normality of the distribution of quantitative variables was verified using the Shapiro–Wilk test. A logistic regression model was used to identify factors significantly influencing older people′s uptake of physical activity and performance of strengthening exercises. The level of statistical significance was set at *p* < 0.05. Data were analyzed using Statistica version 13.1 (TIBCO Software Inc., Palo Alto, CA, USA, 2017, http://statistica.io).

### 2.4. Ethics

The study was conducted in accordance with the Declaration of Helsinki and approved by the Bioethics Committee of the University of Rzeszow (Resolution No. 4/3/2017). All participants provided written informed consent. All methods were performed in accordance with the relevant guidelines and regulations.

## 3. Results

The study included 858 people aged between 75 and 90 years, including 557 women and 301 men. The mean age of the people surveyed was 79.92 (SD = 3.48). Majority of people surveyed lived in a rural area (63.99%) and lived alone (53.96%). The mean pain measured by the VAS scale was 4.23 and the mean number of chronic diseases per person surveyed was 5.56. Most of the elderly did not belong to an organized social group (75.87%) and did not have social contacts (53.50%). About 70% of people indicated that there were communication, architectural and social barriers and obstacles in their living environment. Only 36.83% of respondents were satisfied with their housing conditions. More than half of the people surveyed rated their quality of life as good (50.93%). Only 40% of older people were satisfied with their health. In the surveyed group, only 25.64% of people did a minimum of 150 min of moderate-intensity exercise, while strengthening exercises were performed by 22.49% (Table 1).

Physical activity of at least 150 min per week is significantly more often undertaken by younger people than older people, who are better educated, more socially active, with lower pain levels and fewer chronic diseases. They are also people whose environment had fewer barriers and obstacles than that of physically inactive people, and who rated their health status and quality of life higher (Table 1).

Strengthening exercises are significantly more often performed by people living in urban areas than in rural areas, those living in a relationship, those who are better educated and those with higher incomes. They are also more likely to be socially connected and socially active, to have better relationships with relatives, to live in an environment with fewer barriers and handicaps, to be satisfied with their housing conditions and to rate their health and quality of life higher (Table 1).

Older people undertaking a minimum of 150 min of physical activity per week had lower overall disability and better functioning in: cognitive, mobility, caring for themselves, maintaining good relationships with others and participating in social life. Similar results were obtained by older people performing strengthening exercises (Table 2).

Older people undertaking a minimum of 150 min of PA per week had a higher quality of life, especially in terms of physical and psychological functioning. In contrast, those performing strengthening exercises had a better quality of life in all its domains (Table 3).

Variables that significantly differentiated the study population in terms of undertaking physical activity and performing strengthening exercises were included in the model. An important factor associated with undertaking a minimum of 150 min of PA per week was age. The chance of being physically active was shown to decrease by an average of 7% with each additional year. Another significant factor for undertaking PA was the number of chronic diseases (with each additional disease, the chance of PA decreased by 8%). Those who were socially active were almost 1.5 times more likely to undertake PA. Those who maintained social contacts were more than 2 times more likely to undertake PA compared to those who did not. It was also shown that those who rated their quality of life poorly were 73% less likely to undertake PA than those for whom their quality of life was indifferent. Seniors who were dissatisfied with their health were 40% less likely to undertake PA than those who rated their health as satisfied or very satisfied (Table 4).

An important factor associated with the performance of strengthening exercises by older people was the place of residence. Urban residents were more than 1.8 times more likely to perform this type of exercise than rural residents. Seniors with a secondary education were almost 1.6 times more likely to perform strengthening exercises than those with at most vocational education. Seniors with social contacts were almost 3 times more likely to perform strengthening exercises than others. It was also shown that seniors who were satisfied with the housing conditions associated with their daily living were 1.8 times more likely to undertake PA.

## 4. Discussion

In recent decades, Poland has seen a slowdown in demographic development and significant changes in the age structure of the population associated with dynamic ageing of the population, including, in particular, an increase in the proportion of people over 70 years of age. These changes are typical of all highly developed countries. The elderly population in Poland is dominated by women. In 2020, the share of women among the elderly was 58.1%. The numerical advantage of women over men increases significantly with age and among those aged 75 and over, and women now already account for 66.2%. Most senior citizens live in urban areas. Among urban residents, older people account for approximately 27.8%, and 22.3% of the rural population. Older people in Poland are characterized by low income. One in ten older people in Poland cannot afford to buy all the medicines prescribed by doctors. A characteristic feature is the reduction in the number of multigenerational families and thus the support function of seniors in the family. Apart from the possible feeling of loneliness under such circumstances, which adversely affects the quality of life of older people, single-person households (as well as single-generation households) are less able to independently meet their needs. Regarding the health needs of the elderly in Poland, the primary role in ambulatory care is played by the family doctor and the Primary Healthcare Clinic. The health status of Polish seniors is worse than the average for European Union countries. People of advanced age, with multimorbidity and disabilities, especially those living in rural areas, are unable to reach specialists or benefit from curative rehabilitation [22].

Majority of older people aged 75 to 90 years did not meet the WHO recommendations for undertaking PA. Only 25.64% of older people undertook a minimum of 150 min per week of moderate-intensity PA and only 22.49% performed strengthening exercises. According to a report by the Ministry of Sport and Tourism in Poland, only 30.6% of 15–69-year-olds meet the WHO′s requirements for undertaking PA, and this percentage decreases with age [14]. The Polish Central Statistical Office (CSO) indicates that almost 80% of older people in Poland are physically inactive [23]. In the conducted study, the number of older people meeting the WHO physical activity criteria is lower than that observed in other European studies. In the World Health and SAGE studies, non-compliance with PA recommendations was found in about 30–40% of people aged 70–79 and nearly half of the population aged 80+ [24].

According to a systematic review by Cunningham et al., physically active older people are at reduced risk of death from all causes and cardiovascular disease, breast and prostate cancer, fractures, recurrent falls, ADL disability and functional limitation and cognitive decline, dementia, Alzheimer′s disease and depression. They also experience a healthier ageing process [25], a better quality of life and improved cognitive functioning. Therefore, reducing physical inactivity is becoming one of the most important health practices among the senior community [26,27].

The study found that an important factor associated with undertaking PA was age. The level of PA decreases with age, and the number of steps taken also decreases, resulting in a reduction in total daily activity in people over 65 years of age [28]. This observation is consistent with the findings of studies by other authors in European countries [10,29,30]. Furthermore, Eurobarometer studies show that, overall, the total time spent in a sedentary lifestyle in older people per day has had a significant increasing trend over the years [31].

Another important factor for undertaking PA was the number of chronic diseases. With each additional illness, the chance of undertaking PA decreased by 8%. Similar results have been obtained by other authors, showing that older people with more chronic diseases are more likely to fail to meet the minimum requirements of PA [29]. Health status stands out as one of the main factors for participation in exercise. Different symptoms of medical conditions may influence lower PA values. Chronic diseases that are associated with pain or fatigue may reduce engagement in PA [32]. Older people with depression are also more likely to give up exercise [33]. It is important to properly assess the health status of older people before they start physical activity. There is a growing body of scientific evidence demonstrating the health benefits of supervised physical activity intervention programs for older people in institutional settings and for older people living in the community [34,35]. Awareness campaigns discussing the barriers before starting PA and the benefits of taking it up seem to be important [36].

It was found that older people with a higher level of education had a higher chance of undertaking PA. A similar relationship was shown by Lubas et al. in a cross-sectional and longitudinal study conducted in European countries [30]. Higher levels of PA were indicated in older people with knowledge of the health benefits of physical activity and the availability of PA [37].

It was also shown that people who were active and maintained social contacts were more likely to undertake PA. According to a systematic review by Smith et al., social activity is an important factor in helping older people to start and continue PA. The support of friends and family is important for undertaking PA in leisure time [38]. Promoting the social benefits of participating in leisure-time PA should become part of interventions targeting older people. Consideration should be given to different forms of social support tailored to the needs of the ageing population to be included in interventions that increase participation in PA [39]. The WHO identifies social support and connectedness as a key determinant of active ageing [40,41]. It is important that social interactions are sustained with age, as good social functioning is associated with improved self-efficacy, translating into increased activity in older people [42].

Self-esteem of quality of life was another important variable affecting the chance of undertaking physical activity. Older people with poorer self-esteem were 73% less likely to be physically active. Puciato et al. indicated that PA affects various domains of quality of life. Taking up physical activity by seniors has a preventive and therapeutic effect and improves quality of life compared to inactive people [43]. Improving quality of life is one of the key elements in the development strategies of many countries and regions and improving older people′s engagement in PA is an important aspect of this.

The study also found that seniors who are dissatisfied with their living condition have a 40% lower chance of PA. Previous research has shown that adverse health outcomes are associated with high levels of sedentary lifestyles in older people [44].

Factors related to the performance of strengthening exercises by older people were also assessed. Urban residents were more likely to undertake strengthening exercises. Similar results were also observed in a study by Rowinski et al. [45]. Cities play a key role in enabling older people to live longer and healthier lives. Certain attributes of where people live and urban planning, among others, related to accessibility, can influence the increased PA of older people [46].

In our study, it was shown that older people who were satisfied with their housing conditions were characterized as being more likely to perform strengthening exercises. Barriers present at home may impede mobility in older people. However, attractive destinations located outside the home may correlate with greater PA and potentially motivate people to go outside and exercise [47].

This study has some limitations. Firstly, it was a cross-sectional study which prevents a thorough causal analysis of the links between PA and its determinants. Secondly, specially designed survey questions were used to assess PA. Future studies should also include objective measures to assess PA among older people. The survey is representative of the south-eastern region of Poland and is characterized by a higher proportion of rural residents. Future studies should include a representative sample of older people from across the country.

The strengths of the study lie primarily in the identification of significant, largely modifiable, predictors of physical activity engagement among older people from a large sample of older adults aged 75 and over.

## 5. Conclusions

In conclusion, the study population represented a low level of physical activity uptake, with the majority not meeting the WHO recommendations for physical activity in older people. Considering the current evidence on the benefits of exercise for older people and the results of our study, it seems important to implement a strategy to increase physical activity among older people based on several key pillars:

1. Effective promotional campaigns targeting older people, implemented not only through occasional advertising in the media, but primarily by primary healthcare professionals, especially GPs, who are a profession of public trust in Poland.
2. Introduction of free forms of physical activity for the elderly conducted by physiotherapists, carried out upon the order of a doctor or physiotherapy specialist.
3. Implementation of evidence-based exercise programs for both seniors living in the community and those residing in health and social care institutions.
4. Careful assessment of the living environment of older people, especially in rural environments, to address barriers that prevent older people from maintaining daily physical and social activities.

## Figures and Tables

**Table 1:** Characteristics of the study population of people aged 75–90 years (*n* = 858).

Variables		Total	Physical Activity Min. 150 Min		Performing Strengthening Exercises	
		Number (%) Mean (SD)	Number (%) Mean (SD)	*p*-Value	Number (%) Mean (SD)	*p*-Value
*Socioeconomic*						
Age		79.92 (3.48)	79.22 (3.17)	0.001 ^b^	79.69 (3.27)	0.585 ^b^
Gender	Females	557 (64.92)	139 (24.96)	0.531 ^c^	127 (22.80)	0.770 ^c^
	Males	301 (35.08)	81 (26.91)		66 (21.93)	
Place of residence	Town	309 (36.01)	77 (24.92)	0.716 ^c^	96 (31.07)	<0.001 ^c^
	Village	549 (63.99)	143 (26.05)		97 (17.67)	
Marital status	In relationship	395 (46.04)	110 (27.85)	0.171 ^c^	102 (25.82)	0.031 ^c^
	Single	463 (53.96)	110 (23.76)		91 (19.65)	
Education	At most vocational	593 (69.11)	137 (23.10)	0.011 ^c^	103 (17.37)	<0.001 ^c^
	At least secondary	265 (30.89)	83 (31.32)		90 (33.96)	
Income ^a^	Up to 2000 PLN and less/person	491 (78.81)	133 (27.09)	0.575 ^c^	80 (16.29)	<0.001 ^c^
	2001 PLN and more	132 (21.19)	39 (29.55)		50 (37.88)	
*Physical health*						
ICF B530 BMI ICF B280		26.99 (4.65)	26.70 (4.13)	0.480 ^b^	26.67 (4.07)	0.510 ^b^
Pain on the VAS scale		4.23 (2.76)	3.48 (2.79)	<0.001 ^b^	4.04 (2.83)	0.382 ^b^
Number of chronic diseases		5.56 (3.19)	4.73 (3.31)	<0.001 ^b^	5.66 (3.26)	0.387 ^b^
*Social activity and participation*						
ICF D910 Social activity and participation	No	651 (75.87)	155 (23.81)	0.029 ^c^	134 (20.58)	0.017 ^c^
	Yes	207 (24.13)	65 (29.55)		186 (28.50)	
ICF D720 D760 Maintenance of social contacts	No	459 (53.50)	116 (25.27)	0.791 ^c^	53 (11.55)	<0.001 ^c^
	Yes	399 (46.50)	104 (26.07)		140 (35.09)	
*Environment*						
ICF E155 Presence of barriers and obstacles (including architectural, communication, social and other barriers) in the environment of the respondent	No	258 (30.07)	108 (41.86)	<0.001 ^c^	93 (36.05)	<0.001 ^c^
	Yes	600 (69.93)	340 (18.67)		100 (16.67)	
Assessment of the residential conditions related to the presence of barriers/facilitators to everyday functioning	Unsatisfied/very unsatisfied	209 (24.36)	58 (27.75)	0.633 ^c^	32 (15.31)	<0.001 ^c^
	Neither satisfied/nor unsatisfied	333 (38.81)	282 (25.83)		59 (17.72)	
	Satisfied/very satisfied	316 (36.83)	280 (24.05)		102 (32.28)	
*Self-assessment of quality of life and satisfaction with health status*						
Self-assessment of quality of life	Bad/very bad	84 (9.79)	6 (7.14)	<0.001 ^c^	5 (5.95)	<0.001 ^c^
	Neither good nor bad	337 (39.28)	78 (23.15)		60 (17.80)	
	Good/very good	437 (50.93)	136 (31.12)		128 (29.29)	
Self-assessment of satisfaction with health status	Unsatisfied/very unsatisfied	259 (30.19)	34 (13.13)	<0.001 ^c^	40 (15.44)	<0.001 ^c^
	Neither satisfied nor unsatisfied	729 (33.03)	74 (26.71)		53 (19.13)	
	Satisfied/very satisfied	901 (40.82)	112 (34.78)		100 (31.06)	

^a^ The lack of data of 623 people. ^b^ Mann–Whitney test. ^c^ Chi-square test.

**Table 2:** Characteristics level of disability of people aged 75–90 years (*n* = 858).

Variables	Total	Physical Activity Min. 150 min		Performing Strengthening Exercises	
WHODAS 2.0	Number (%) Mean (SD)	Number (%) Mean (SD)	*p*-Value	Number (%) Mean (SD)	*p*-Value
Do1. Cognition	30.29 (22.38)	25.84 (19.07)	0.002 ^b^	17.54 (18.76)	<0.001 ^b^
Do2. Mobility	45.13 (29.12)	32.76 (23.61)	<0.001 ^b^	37.76 (30.16)	<0.001 ^b^
Do3. Self-care	23.23 (25.76)	14.41 (19.40)	<0.001 ^b^	15.96 (23.14)	<0.001 ^b^
Do4. Getting along	33.19 (26.84)	30.95 (25.33)	0.219 ^b^	17.49 (20.28)	<0.001 ^b^
Do5. Life activities	47.65 (29.98)	37.23 (24.90)	<0.001 ^b^	35.80 (30.01)	<0.001 ^b^
Do6. Participation	35.51 (25.33)	32.88 (23.84)	0.043 ^b^	33.05 (24.47)	0.173 ^b^
Total disability	35.73 (18.58)	29.54 (14.74)	<0.001 ^b^	26.91 (17.17)	<0.001 ^b^

^b^ Mann–Whitney test.

**Table 3:** Characteristics of quality of life of people aged 75–90 years (*n* = 858).

Variables	Total	Physical Activity Min. 150 min		Performing Strengthening Exercises	
WHOQOL-Brief Domains	Number (%) Mean (SD)	Number (%) Mean (SD)	*p*-Value	Number (%) Mean (SD)	*p*-Value
Physical functioning	52.55 (15.21)	58.32 (13.95)	<0.001 ^b^	57.73 (15.2)	<0.001 ^b^
Psychological functioning	59.94 (15.24)	61.82 (14.91)	0.038 ^b^	66.59 (15.21)	<0.001 ^b^
Social relations	65.67 (16.01)	66.97 (15.49)	0.206 ^b^	71.98 (15.37)	<0.001 ^b^
Environment	60.92 (15.03)	62.68 (15.66)	0.066 ^b^	68.51 (15.06)	<0.001 ^b^

^b^ Mann–Whitney test.

**Table 4:** Logistic regression models illustrating factors influencing the undertaking of physical activity, min. 150 min/week, and performing strengthening exercises of people aged 75–90 years (*n* = 858).

Variables			Physical Activity Min. 150 min			Performing Strengthening Exercises	
		Odds Ratio	95% CI	*p*-Value	Odds Ratio	95% CI	*p*-Value
Age		0.93	(0.88–0.98)	0.007	-	-	-
Place of residence, (reference village) town		-	-	-	1.84	(1.24–2.73)	0.003
Marital status, (reference single) in relationship		-	-	-	1.28	(0.90–1.83)	0.173
Education, (reference at most vocational) at least secondary level		1.20	(0.85–1.71)	0.305	1.57	(1.05–2.34)	0.027
*Physical health*							
ICF B280 Pain on the VAS scale		0.99	(0.92–1.08)	0.978	-	-	-
Number of diseases		0.92	(0.86–0.98)	0.001	-	-	-
*Social activity and participation*							
ICF D910 Social activity and participation, (reference no) yes		1.47	(1.01–2.14)	0.044	1.28	(0.86–1.92)	0.225
ICF D720 D760 Maintenance of social contacts, (reference no) yes		2.04	(1.41–2.97)	< 0.001	2.89	(1.95–4.29)	<0.001
*Environment*							
ICF E155 Presence of barriers and obstacles (including architectural, communication, social and other barriers) in the environment of the respondent, (reference yes) no		2.30	(1.61–3.29)	<0.001	1.23	(2.32–3.83)	0.318
Assessment of the residential conditions related to the presence of barriers/facilitators to everyday functioning, (reference neither satisfied nor unsatisfied)	Unsatisfied/very unsatisfied	-	-	-	0.95	(0.57–1.58)	0.850
	Satisfied/very satisfied	-	-	-	1.80	(1.21–2.70)	0.004
*Self-assessment of quality of life and satisfaction with health status*							
Self-assessment of quality of life (reference neither good nor bad)	Bad/very bad	0.37	(0.15–0.94)	0.035	0.52	(0.19–1.41)	0.199
	Good/very good	0.93	(0.63–1.36)	0.694	1.23	(0.81–1.86)	0.327
Self-assessment of satisfaction with health, (reference neither satisfied nor unsatisfied)	Unsatisfied/very unsatisfied	0.60	(0.37–0.99)	0.047	1.06	(0.64–1.75)	0.327
	Satisfied/very satisfied	1.02	(0.68–1.51)	0.940	1.54	(0.99–2.38)	0.051

## Data Availability

The datasets used and analyzed during the current study are available from the corresponding author upon reasonable request.

## References

[1] Mendis S. (2014). Global Status Report on Noncommunicable Diseases 2014 [Internet].

[2] Hallal P.C., Andersen L.B., Bull F.C., Guthold R., Haskell W., Ekelund U. (2012). Global physical activity levels: Surveillance progress, pitfalls, and prospects. Lancet.

[3] (2017). Uczestnictwo w Sporcie i Rekreacji Ruchowej w 2016 r.

[4] Chodzko-Zajko W.J., Proctor D.N., Singh M.A.F., Minson C.T., Nigg C.R., Salem G.J., Skinner J.S. (2009). American College of Sports Medicine position stand. Exercise and physical activity for older adults. Med. Sci. Sport. Exerc..

[5] Paterson D.H., Warburton D.E. (2010). Physical activity and functional limitations in older adults: A systematic review related to Canada’s Physical Activity Guidelines. Int. J. Behav. Nutr. Phys. Act..

[6] Stessman J., Hammerman-Rozenberg R., Cohen A., Ein-Mor E., Jacobs J.M. (2009). Physical activity, function, and longevity among the very old. Arch. Intern. Med..

[7] Kleinke F., Penndorf P., Ulbricht S., Dörr M., Hoffmann W., van den Berg N. (2020). Levels of and determinants for physical activity and physical inactivity in a group of healthy elderly people in Germany: Baseline results of the MOVING-study. PLoS ONE.

[8] World Health Organization (2010). Global Recommendations on Physical Activity for Health.

[9] Sun F., Norman I.J., While A.E. (2013). Physical activity in older people: A systematic review. BMC Public Health.

[10] Bauman A., Merom D., Bull F.C., Buchner D.M., Fiatarone Singh M.A. (2016). Updating the evidence for physical activity: Summative reviews of the epidemiological evidence, prevalence, and interventions to promote “active aging”. Gerontologist.

[11] Bauman A.E., Reis R.S., Sallis J.F., Wells J.C., Loos R.J.F., Martin B.W. (2012). Correlates of physical activity: Why are some people physically active and others not?. Lancet.

[12] Lee H.J., Lee Y., Yun J. (2020). Factors Associated with Physical Activity in Older Adults by Region: Based on the 2017 Community Health Survey. J. Korean Acad. Community Health Nurs..

[13] McNeill L.H., Kreuter M.W., Subramanian S.V. (2006). Social environment and physical activity: A review of concepts and evidence. Soc. Sci. Med..

[14] Poziom Aktywności Fizycznej Polaków 2018. https://www.gov.pl/web/sport/aktywnosc-fizyczna-spoleczenstwa2.

[15] Piotrowicz K., Romanik W., Skalska A., Gryglewska B., Szczerbińska K., Derejczyk J., Krzyżewski R.M., Grodzicki T., Gąsowski J. (2019). The comparison of the 1972 Hodkinson’s Abbreviated Mental Test Score (AMTS) and its variants in screening for cognitive impairment. Aging Clin. Exp. Res..

[16] European Network for Action on Ageing and Physical Activity—EUNAAPA. http://www.eunaapa.org.

[17] World Health Organization (2011). ICF. Disability and Health.

[18] Stucki G., Pollock A., Engkasan J.P., Selb M. (2019). How to use the ICF as a reference system for comparative evaluation and standardised reporting of rehabilitation interventions. Eur. J. Phys. Rehabil. Med..

[19] Üstün T.B., Kostanjsek N., Chatterji S., Rehm J. (2001). Measuring Health and Disability: Manual for WHO Disability Assessment Schedule (WHODAS 2.0).

[20] WHOQOL: Measuring Quality of Life. https://www.who.int/tools/whoqol.

[21] Skevington S.M., Lotfy M. (2004). WHOQOL Group. The World Health Organization’s WHOQOL-BREF quality of life assessment: Psychometric properties and results of the international field trial. A report from the WHOQOL group. Qual. Life Res..

[22] Błędowski P., Grodzicki T., Mossakowska M., Zdrojewski T., PolSenior2 (2021). Badanie Poszczegόlnych Obszarόw Stanu Zdrowia osόb Starszych, w tym Jakości życia Związanej ze Zdrowiem, Red.

[23] GUS (2009). Informacje i Opracowania Statystyczne: Uczestnictwo Polaków w Sporcie i Rekreacji Ruchowej.

[24] Guthold R., Stevens G.A., Riley L.M., Bull F.C. (2018). Worldwide trends in insufficient physical activity from 2001 to 2016: A pooled analysis of 358 population-based surveys with 1.9 million participants. Lancet Glob. Health.

[25] Cunningham C., O’ Sullivan R., Caserotti P., Tully M.A. (2020). Consequences of physical inactivity in older adults: A systematic review of reviews and meta-analyses. Scand. J. Med. Sci. Sport..

[26] Wu C.Y., Miller L.M., Wall R.N., Beattie Z.T., Silbert L.C., Kaye J.A. (2020). Prolonged Physical Inactivity in Older Adult Couples: A Dyadic Analysis Using Actigraphy. Innov. Aging.

[27] Moreno-Agostino D., Daskalopoulou C., Wu Y.T., Koukounari A., Haro J.M., Tyrovolas S., Panagiotakos D.B., Prince M., Prina A.M. (2020). The impact of physical activity on healthy ageing trajectories: Evidence from eight cohort studies. Int. J. Behav. Nutr. Phys. Act..

[28] Takagi D., Nishida Y., Fujita D. (2015). Age-associated changes in the level of physical activity in elderly adults. J. Phys. Ther. Sci..

[29] Mattle M., Meyer U., Lang W., Mantegazza N., Gagesch M., Mansky R., Kressig R.W., Egli A., Orav E.J., Bischoff-Ferrari H.A. (2022). Prevalence of Physical Activity and Sedentary Behavior Patterns in Generally Healthy European Adults Aged 70 Years and Older-Baseline Results from the DO-HEALTH Clinical Trial. Front. Public Health.

[30] Lubs L., Peplies J., Drell C., Bammann K. (2018). Cross-sectional and longitudinal factors influencing physical activity of 65 to 75-year-olds: A pan European cohort study based on the survey of health, ageing and retirement in Europe (SHARE). BMC Geriatr..

[31] Lopez-Valenciano A., Mayo X., Liguori G., Copeland R.J., Lamb M., Jimenez A. (2020). Changes in sedentary behaviour in European Union adults between 2002 and (2017). BMC Public Health.

[32] Catala P., Lopez-Roig S., Ecija C., Suso-Ribera C., Peñacoba Puente C. (2021). Why do some people with severe chronic pain adhere to walking prescriptions whilst others won’t? A cross-sectional study exploring clinical and psychosocial predictors in women with fibromyalgia. Rheumatol. Int..

[33] Bachmann C., Oesch P., Bachmann S. (2017). Recommendations for improving adherence to home-based exercise: A systematic review. Phys. Med. Rehabil. Kurortmed..

[34] Warburton D.E.R., Bredin S.S.D. (2017). Health benefits of physical activity: A systematic review of current systematic reviews. Curr. Opin. Cardiol..

[35] Izquierdo M., Duque G., Morley J.E. (2021). Physical activity guidelines for older people: Knowledge gaps and future directions. Lancet Healthy Longev..

[36] Collado-Mateo D., Lavín-Pérez A.M., Peñacoba C., Del Coso J., Leyton-Román M., Luque-Casado A., Gasque P., Fernández-del-Olmo M.Á., Amado-Alonso D. (2021). Key Factors Associated with Adherence to Physical Exercise in Patients with Chronic Diseases and Older Adults: An Umbrella Review. J. Environ. Res. Public Health.

[37] Notthoff N., Reisch P., Gerstorf D. (2017). Individual characteristics and physical activity in older adults: A systematic review. Gerontology.

[38] Lindsay Smith G., Banting L., Eime R., O’Sullivan G., van Uffelen J.G.Z. (2017). The association between social support and physical activity in older adults: A systematic review. Int. J. Behav. Nutr. Phys. Act..

[39] Zimmer C., McDonough M.H. (2022). Social Support and Physical Activity in Older Adults: Identifying Predictors Using Data from the Canadian Longitudinal Study on Aging. J. Aging Phys. Act..

[40] World Health Organization (2002). Active Ageing: A Policy Framework.

[41] World Health Organization Decade of Healthy Ageing 2020–2030. https://cdn.who.int/media/docs/default-source/decade-of-healthy-ageing/final-decade-proposal/decade-proposal-final-apr2020-en.pdf?sfvrsn=b4b75ebc_25&download=true.

[42] Carlson J.A., Sallis J.F., Conway T.L., Saelens B.E., Frank L.D., Kerr J., Cain K.L., King A.C. (2012). Interactions between psychosocial and built environment factors in explaining older adults’ physical activity. Prev. Med..

[43] Puciato D., Borysiuk Z., Rozpara M. (2017). Quality of life and physical activity in an older working-age population. Clin. Interv. Aging.

[44] Ekelund U., Steene-Johannessen J., Brown W.J., Fagerland M.W., Owen N., Powell K.E., Bauman A., Lee I.M., Series L.P.A. (2016). Does physical activity attenuate, or even eliminate, the detrimental association of sitting time with mortality? A harmonised meta-analysis of data from more than 1 million men and women. Lancet.

[45] Rowiński R., Kowalska G., Kozakiewicz M., Kędziora-Kornatowska K., Kornatowski M., Hawlena J., Rowińska K. (2021). Physical Activity and Its Determinants among Senior Residents of Podlasie, a Green Region of Poland, Based on the National PolSenior Study. Int. J. Environ. Res. Public Health.

[46] Ellis G., Hunter R.F., Hino A.A.F., Cleland C.L., Ferguson S., Murtagh B., Anez C.R.R., Melo S., Tully M., Kee F. (2018). Study protocol: Healthy urban living and ageing in place (HULAP): An international, mixed methods study examining the associations between physical activity, built and social environments for older adults the UK and Brazil. BMC Public Health.

[47] Portegijs E., Keskinen K.E., Eronen J., Saajanaho M., Rantakokko M., Rantanen T. (2020). Older Adults’ Physical Activity and the Relevance of Distances to Neighborhood Destinations and Barriers to Outdoor Mobility. Front. Public Health.

